# Biochemical and Histological Evidence on the Protective Effects of *Allium hirtifolium Boiss* (Persian Shallot) as an Herbal Supplement in Cadmium-Induced Hepatotoxicity

**DOI:** 10.1155/2020/7457504

**Published:** 2020-06-17

**Authors:** Navid Omidifar, Amir Nili-Ahmadabadi, Ahmad Gholami, Dara Dastan, Davoud Ahmadimoghaddam, Hossein Nili-Ahmadabadi

**Affiliations:** ^1^Biotechnology Research Center, Department of Pathology, School of Medicine, and Clinical Education Research Center, Zeinabieh Hospital, Shiraz University of Medical Sciences, Shiraz, Iran; ^2^Medicinal Plants and Natural Products Research Center, Hamadan University of Medical Sciences, Hamadan, Iran; ^3^Department of Pharmacology and Toxicology, School of Pharmacy, Hamadan University of Medical Sciences, Hamadan, Iran; ^4^Pharmaceutical Sciences Research Center, Shiraz University of Medical Sciences, Shiraz, Iran; ^5^Department of Pharmacognosy, School of Pharmacy, Hamadan University of Medical Sciences, Hamadan, Iran; ^6^Department of Internal Medicine and Gastroenterology, School of Medicine, Yasuj University of Medical Sciences, Yasuj, Iran

## Abstract

**Materials and Methods:**

Thirty-six male Wistar rats were divided into six groups: groups 1, 2, and 3 received vehicle, Cd (100 mg/L/day by drinking water), and *A. hirtifolium* extract (200 mg/kg/day; orally), respectively. Groups 4, 5, and 6 were Cd groups which were treated with *A. hirtifolium* extract (50, 100, and 200 mg/kg/day, respectively). After 2 weeks, liver enzymes such as alanine aminotransferase (ALT), aspartate aminotransferase (AST), and alkaline phosphatase (ALP) and also oxidative stress biomarkers including lipid peroxidation (LPO), total antioxidant capacity (TAC), total thiol molecule (TTM), and the histopathological changes were determined using standard procedure.

**Results:**

The findings showed that Cd caused a remarkable rise in levels of serum hepatic enzymes such as ALT (*P* < 0.001), AST (*P* < 0.01) and ALP (*P* < 0.001) compared with the control group. In addition, Cd led to the decreasing of the levels of TTM (*P* < 0.001) and TAC (*P* < 0.001) and increasing of LPO (*P* < 0.001) in liver tissue in comparison with the control group. In this regard, remarkable vascular congestion, hepatocellular degeneration, and vacuolization were observed in hepatic tissue of Cd-treated rats. Following the administration of *A. hirtifolium* extract, a significant improvement was observed in the functional and oxidative stress indices of hepatic tissue alongside histopathologic changes.

**Conclusion:**

The current study indicated that the *A. hirtifolium* extract might prevent hepatic oxidative injury by improving oxidant/antioxidant balance in rats exposed to Cd.

## 1. Introduction

*Allium hirtifolium Boiss,* known as Persian shallot “Mooseer,” grows wild as blackish, paper-like tunics in some areas of Iran and other Asian countries. It is traditionally used in the routine diet in countries of central Asia as a spice and flavoring agent in food, especially yoghurt. Recent research has suggested that *A. hirtifolium* is composed of 9-hexadecenoic acid, 11,14-eicosadienoic acid, and n-hexadecanoic acid, and its hydromethanolic extract has strong antimicrobial properties against several bacteria [1]. Allicin (diallyl thiosulfinate) is considered responsible for the antibacterial, antifungal, and antiparasite potentials of *A. hirtifolium* [2]. In addition to the antimicrobial property, the phenolic compound of the ethanol extract of *A. hirtifolium* is reported to have moderate to good antioxidant activity [3, 4], as well as immunomodulatory activity [5] and accelerates wound healing by increasing the epithelialization rate [6]. The active compound of *A. hirtifolium*, shallomin, is also found safe in humans; therefore, research is continued on the various effects of *A. hirtifolium* in different organs [7].

Special attention has been paid to the effect of *A. hirtifolium* on liver protection in animal studies. *A. hirtifolium* extract has shown protective effects against liver cell apoptosis by inhibiting the growth of human hepatoma cancer cells and BCL2 gene [8]. Also, the chloroformic extract of *A. hirtifolium* is found to be able to inhibit proliferation of tumor cell lines including cervical cancer, breast, adenocarcinoma, and connective cell lines [9]. Hydroalcoholic extract of *A. hirtifolium* can also protect rat liver cells against the effects of oxidants in alloxan-induced diabetes and reduce the serum concentrations of aspartate aminotransferase (AST), alanine aminotransferase (ALT), alkaline phosphatase (ALP), and lactate dehydrogenase (LDH) [10, 11]. The favorable result of the hydroalchoholic extract of *A. hirtifolium* on reducing the serum blood glucose levels, glucokinase activity, and gene expression has also been suggested by other researchers [12]. Nevertheless, in another study, it has been shown that *A. hirtifolium* extract could not decrease serum levels of apo-lipoproteins, AST, ALT, glucose, or insulin, while it could significantly reduce serum levels of triglyceride and cholesterol as well as atherosclerotic plaque thickness to media [13]. Accordingly, the efficacy of *A. hirtifolium* on liver enzymes is controversial, and further research is required in this regard.

Cadmium (Cd) is a food contaminant that increases the incidence rate of liver disease [14, 15] and induces oxidative stress in different body organs, especially liver, kidney, and blood, by generating reactive oxygen species (ROS) and impairing the antioxidant defense system [16]. Accordingly, this metal is used to induce liver failure in different animals [17]. Therefore, the aim of the study was to evaluate the therapeutic potential of *A. hirtifolium* in Cd-induced hepatotoxicity in rats.

## 2. Materials and Methods

### 2.1. Preparation of the Extract

The bulbs of the *A. hirtifolium* were collected from Hamadan in western Iran. The plant was identified by the herbarium section of School of Pharmacy, Hamadan University of Medical Sciences (HUMS), Hamadan, Iran, with code number 234. The bulbs were ground and extracted by methanol/water (50 : 50) at 25°C for 48 h in triplicate using a maceration method. Then, the extract was filtrated and evaporated to become dry in a rotary evaporator (Heidolph, Germany) under vacuum at 40°C. The resulting extract was kept in a dark place at 4°C.

### 2.2. Phytochemical Screening

The phytochemical analyses were performed on the *A. hirtifolium* extract using the standard methods to identify secondary metabolites such as alkaloids, saponins, tannins, flavonoids, steroids, terpenoids, proteins, amino acids, glycosides, and anthraquinones [18, 19].

### 2.3. Determination of Total Phenolic Content

The total phenolic content of the *A. hirtifolium* extract was determined according to the Folin–Ciocalteu procedure, 2 hours and 1, 7, and 14 days after the extraction (each experiment was performed in triplicate). The results were expressed as mg gallic acid equivalents (GAE)/g extract [20].

### 2.4. Determination of Total Flavonoid Content

Total flavonoid content of the *A. hirtifolium* extract was determined based on the method reported by Fathollahi et al. [21], 2 hours and 1, 7, and 14 days after the extraction (each experiment was performed in triplicate). The results were expressed as mg quercetin/g extract [21].

### 2.5. Animals and Experimental Design

Thirty-six adult male Wistar rats, weighted 210–240 g, were obtained from the animal house of Hamadan University of Medical Sciences (HUMS), kept in polypropylene cages at room temperature (25 ± 2°C), 12 h dark/12 h light cycle, and humidity of about 50%, and provided with free food and water.

After one week of acclimatization, the animals were divided randomly into six groups and treated as follows:

  Group 1: the rats received normal saline (control group)
  Group 2: the rats received 100 mg/L/day Cd chloride (Cd group)
  Group 3: the rats received 200 mg/kg *A. hirtifolium* extract, orally (*AhB*200)
  Group 4: the rats received 100 mg/L/day Cd chloride by drinking water + 50 mg/kg *A. hirtifolium* extract orally (*AhB*50 + Cd)
  Group 5: the rats received 100 mg/L/day Cd chloride by drinking water + 100 mg/kg *A. hirtifolium* extract orally (*AhB*100 + Cd)
  Group 6: the rats received 100 mg/L/day Cd chloride by drinking water + 200 mg/kg *A. hirtifolium* extract orally (*AhB*200 + Cd)

After 2 weeks of treating animals of the six groups as described above, the animals were anaesthetized by 50 mg/kg ketamine and 10 mg/kg xylazine. The serum samples, obtained by centrifugation of the heart's blood for 15 min at 5000 rpm, were kept at −20°C. A section of the fresh liver was separated and stored at −80°C for biochemical analysis. A part of rat's liver was excised and put in 10% formalin for histological analysis.

### 2.6. Liver Function Experiments

The serum samples were used for examination of ALT, AST, and ALP using colorimetric biochemical kits (Pars Azmon, Iran).

### 2.7. Preparation of Hepatic Tissue Homogenate

The hepatic tissue (100 mg) was homogenized with 1 mL phosphate-buffered saline (50 mM, pH 7.3) and centrifuged at 3000 g for 10 min at 4°C. The supernatant was separated for the biochemical analysis [22].

### 2.8. Measurement of Lipid Peroxidation

The hepatic lipid peroxidation (LPO) was evaluated by determining bioactive aldehydes using the thiobarbituric acid reactive substances (TBARS) method [23]. In brief, 100 *µ*l of hepatic tissue homogenate was mixed with 500 *µ*l reagent containing 2-thiobarbituric acid (TBA, 0.2%) in H_2_SO_4_ (0.05 M). The mixture was heated for 30 min at 100°C in boiling water bath. Then, the optimum absorbance was measured at 532 nm against different concentrations of malondialdehyde (MDA) as the standard, and its findings were reported as nmol/mg protein.

### 2.9. Measurement of Total Antioxidant Capacity

The total antioxidant capacity of tissue homogenate was measured by determining its ability to reduce Fe^+3^ to Fe^+2^ using the ferric-reducing antioxidant power (FRAP) method [24]. In brief, FRAP reagent was prepared by mixing 1 volume of 20 mM FeCl_3_, 10 volumes of 300 mM acetate buffer (pH 3.6), and also 1 volume of 10 mM 2,4,6-tripyridyl-s-triazine (TPTZ) in 40 mM HCL. The complex between Fe^2+^ and TPTZ, as an indicator, gives a blue color with an absorbance maximum at 593 nm. The results were presented as nmol/mg protein.

### 2.10. Measurement of Total Thiol Molecules

Total thiol molecules (TTM) were assayed using 5,5′dithiobis-2-nitro benzoic acid (DTNB) as the reagent, and its absorbance was read against a blank at 412 nm [25]. 200 *µ*l of tris-EDTA buffer solution (0.25 M tris base and 20 mM EDTA, pH 8.2) was mixed with 10 *µ*l of homogenized tissue, and its optimum absorbance was detected at 412 nm. Then, 10 *µ*l of DTNB solution (10 mmol/l in methanol) was added to each sample and incubated at 37°C for 15 min. The absorbance of the samples (A2) and also DTNB blank (B) was read again at 412 nm. The level of thiol molecules was determined by reduced glutathione as standard and reported as *µ*M/mg protein.

### 2.11. Protein Assay

At the end of each experiment, protein levels were measured in the crude homogenate of hepatic tissues by Bradford method.

### 2.12. Histopathologic Examination

After fixation of hepatic tissue in formalin (10%), the paraffin-embedded block was prepared and cut into 4 *µ*m thick sections using a rotary microtome. The samples were stained with hematoxylin and eosin (H&E) dye for histopathological examination.

### 2.13. Statistical Analysis

All variables were quantitative. The mean values with standard error mean (SEM) were reported for each variable and compared among the six study groups using one-way ANOVA with Tukey's post hoc test. For the statistical analysis, the GraphPad Prism statistical software version 6.0 was used. *P* values <0.05 were considered statistically significant.

## 3. Results

### 3.1. Phytochemical Analysis

Using the phytochemical screening, we obtained that the hydroalcoholic extract of the *A. hirtifolium* is composed of different compounds such as phenols, flavonoids, saponins, glycosides, steroids, tannins, terpenoids, and amino acids (Table 1). The total phenolic and flavonoid contents of *A. hirtifolium* extract were 80.1 ± 0.9 mg GAE/g extract and 58.2 ± 0.4 mg quercetin/g extract, respectively. During the 14 days after the extraction, no significant changes were observed in the phenolic and flavonoid values, which could indicate the stability of extract during this period (data not shown).

### 3.2. The Effects of *A. hirtifolium* Extract on Liver Function

**Figure 1:**
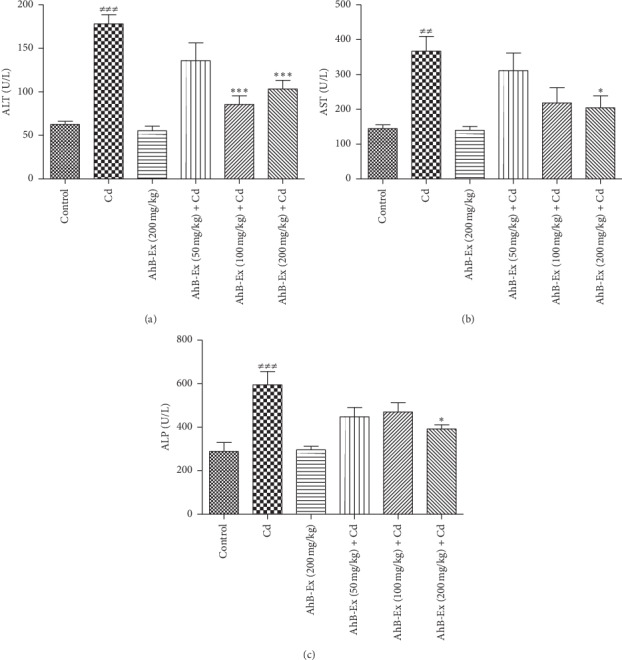
Effects of *Allium hirtifolium Boiss* extract on liver function biomarkers of cadmium*-*exposed rats. Values are expressed as mean ± SEM and compared between the groups (6 rats in each group) using one-way ANOVA with Tukey's post hoc test. ^##^*P* < 0.01 and ^###^*P* < 0.001 vs. control group; ^*∗*^*P* < 0.05 and ^*∗∗∗*^*P* < 0.01 vs. cadmium group. Cadmium was administered by drinking water (100 mg/L) for 2 weeks. Cd: cadmium, AhB-Ex: *Allium hirtifolium Boiss* extract, ALT: alanine aminotransferase, AST: aspartate aminotransferase, and ALP: alkaline phosphatase.

The results of comparing liver enzymes among the six study groups are shown in Figure 1. As illustrated, ALT, AST, and ALP levels of the Cd group were significantly higher than the control group (*P* < 0.001, *P* < 0.01, and *P* < 0.001, respectively). The two pretreatment groups, receiving 100 and 200 mg *A. hirtifolium* extract, had a significantly lower ALT level compared with the Cd group (*P* < 0.001). The *A. hirtifolium* extract could significantly decrease the serum levels of AST and ALP at the dose of 200 mg/kg, compared with the Cd group (*P* < 0.05).

### 3.3. The Effects of *A. hirtifolium* Extract on Hepatic Oxidative Damage

**Figure 2:**
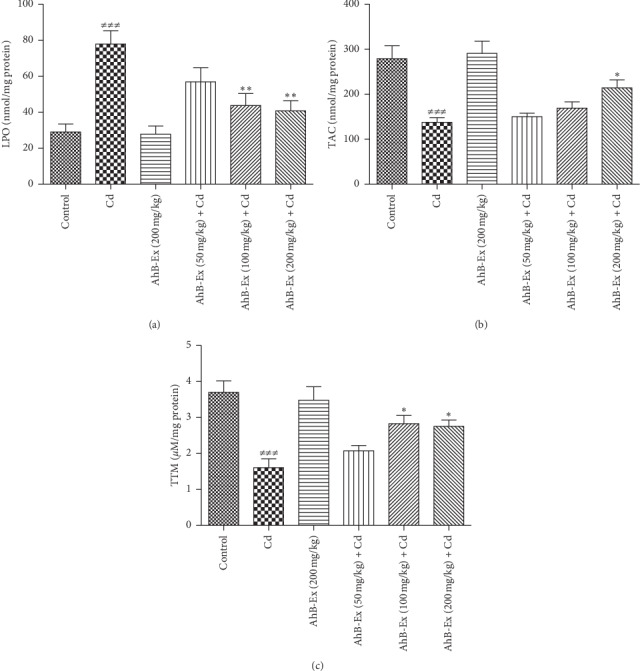
Effects of *Allium hirtifolium Boiss* extract on oxidative stress biomarkers in the liver of cadmium-exposed rats. Values are expressed as mean ± SEM and compared between the groups (6 rats in each group) using one-way ANOVA with Tukey's post hoc test. ^###^*P* < 0.001 vs. control group; ^*∗*^*P* < 0.05 and ^*∗∗*^*P* < 0.01 vs. cadmium group. Cadmium was administered by drinking water (100 mg/L) for 2 weeks. Cd: cadmium, AhB-Ex: *Allium hirtifolium Boiss* extract, LPO: lipid peroxidation, TAC: total antioxidant capacity, and TTM: total thiol molecule.

The results of comparing oxidative stress biomarkers among the six study groups are shown in Figure 2. As illustrated, the Cd group had a significantly higher LPO and lower TAC and TTM than the control group (*P* < 0.001). The two treatment groups, receiving 100 and 200 mg *A. hirtifolium* extract, had a significantly lower LPO level, compared with the Cd group (*P* < 0.01). The *A. hirtifolium* extract could significantly decrease the hepatic LPO level (*P* < 0.01) and increase TTM levels (*P* < 0.05) at the doses of 100 and 200 mg/kg, compared with the Cd group (*P* < 0.01). In addition, a significant increase was observed in the hepatic TAC levels at the dose of 200 mg/kg (*P* < 0.05).

### 3.4. The Effects of *A. hirtifolium* Extract on Pathological Changes

**Figure 3:**
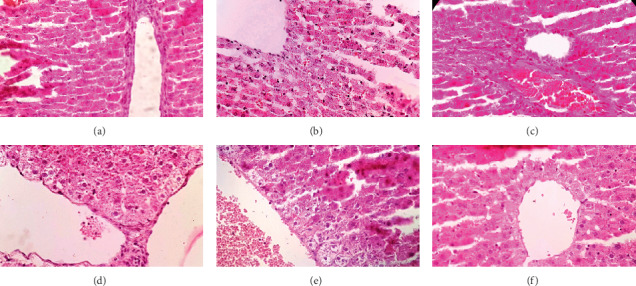
Effects of *Allium hirtifolium Boiss* extract on hepatic tissue of cadmium-exposed rats. The histopathological changes such as vascular congestion and accumulations of inflammatory cells were evaluated in different groups. (a) The control group showing normal appearance of hepatocytes. (b) The cadmium group showing vascular congestion in remarkable degree, hepatocellular degeneration, and vacuolization. (c) The *A. hirtifolium* extract (200 mg/kg) group showing normal appearance of hepatocytes. (d) The cadmium plus *A. hirtifolium* extract (50 mg/kg) group showing rare inflammatory cells, but vascular congestion and cellular degeneration persist. (e) The cadmium plus *A. hirtifolium* extract (100 mg/kg) group showing mild cellular degeneration without obvious inflammation, but congestion still persists. (f) The cadmium plus *A. hirtifolium* extract (200 mg/kg) group showed no pathological changes. Original magnification of all images is ×40.

As mentioned in Figure 3, remarkable vascular congestion, hepatocellular degeneration, and vacuolization were observed in hepatic tissue of Cd-treated rats. Following the pretreatment with different doses of *A. hirtifolium* extract, a remarkable improvement was found in some of the pathological alterations such as vascular congestion and hepatocellular degeneration. The optimum protective effect was observed at the dose of 200 mg/kg of the extract.

## 4. Discussion

The results of this study emphasize the nutritional importance of *A. hirtifolium Boiss* in Cd-induced liver failure. Preliminary phytochemical studies showed significant amounts of phenolic and flavonoid compounds in the *A. hirtifolium* extract. Due to the remarkable antioxidant properties of these compounds, quantitative contents of phenols and flavonoids were determined in the extract. Based on the received dosage range of the *A. hirtifolium* extract for each animal (50–200 mg/kg/day), the minimum and maximum amounts received for phenolic compounds (4–16 mg/kg/day) and flavonoid compounds (2.5–10 mg/kg/day) were estimated.

In the present study, increased hepatic serum enzymes (ALT, AST, and ALP) are considered as an important biomarker in the diagnosis of liver failure [26]. Therefore, the results of the current study indicate disruption of hepatic cells' integrity caused by Cd and occurrence of hepatic failure. Several animal studies have also suggested increased levels of hepatic enzymes in the rats' serum that received Cd chloride in drinking water [27, 28], which confirm the results of the present study and emphasize on the importance of exposure to Cd. Clinical studies have also confirmed the significance of hepatotoxicity induced by Cd, which is present in soil, water, food (dietary intake), and air (smoking and air pollution), and considered an important health concern [15, 29–31]. Other studies have also shown the positive correlation of serum Cd concentrations with liver enzyme levels (ALT, AST, and ALP) in Korean adults [32] and Indonesian pregnant women [33], which is consistent with this study. According to the evidence, the body has a limited capacity to Cd exposure, and the long-term exposure to Cd accumulates Cd and causes toxicity in several organs, such as liver and kidneys and impair their function [34–36], indicating the toxic effect of Cd in different organ systems [37].

Since oxidative stress plays a fundamental role in Cd-induced hepatic failure, the oxidant/antioxidant markers were evaluated in this survey. In the current study, a significant increase in LPO and a noticeable decrease in TTM and TAC liver levels were found in the Cd group, indicating the induction of oxidative damage due to Cd toxicity. Although the accurate mechanism of the increased oxidative stress is still not well understood, review studies have indicated that Cd increases the LPO by changing the intracellular glutathione levels, induces the production of ROS and nicotinamide adenine dinucleotide phosphate (NADPH) oxidase, and inhibits antioxidants [38, 39], which are consistent with the results of the present study.

Due to the significance of oxidative damage caused by Cd, researchers have studied several herbal medicines that can protect against the destructive effects of Cd in the liver with a great focus on oxidative stress [40, 41]. However, none of previous studies have studied the protective effects of *A. hirtifolium* extract on Cd-induced hepatotoxicity.

Another important finding of the present study, considered the main objective of our study, was the effect of *A. hirtifolium* on oxidative damage induced by cadmium. Our findings indicated that *A. hirtifolium* could improve hepatic oxidative damage by increasing the hepatic thiol group and, subsequently, decrease liver LPO. In this regard, the previous studies have suggested that administration of some *Allium* species such as *A. tripedale* and *A. jesdianum* can prevent the effects of hepatotoxic agents such as acetaminophen in rats [42, 43]. *A. hirtifolium* is reported to have the highest antioxidant property, compared with other 12 plant extracts, as well as significant radical scavenging property [44]. This plant and other garlic compounds have a high concentration of sulphur compounds (such as diallyl sulfide, S-ethylcysteine, diallyl disulfide, and N-acetylcysteine) with a strong antioxidant activity that protect against cell injury [45]. *A. hirtifolium* is also reported to have a high concentration of saponins, sulphur-containing compounds, and flavonoids which are responsible for its antioxidant activity [46]. Apparently, the different compositions of the plant extract, obtained from different parts of the country, can cause different degrees of antioxidative and liver-protective effects [4]. In addition, the methanolic extract of *A. hirtifolium* is reported to have a higher concentration of polyphenolic, flavonoid, and proanthocyanidin compounds and a stronger antioxidative property than its aqueous extract [47]. Therefore, the essential components of *A. hirtifolium* are important factors in determining its protective effects.

In conclusion, the present study showed that Cd induced significant liver injury and oxidative stress in rats, and oral administration of 100 and 200 mg *A. hirtifolium* extract could significantly reduce the destructive effect of Cd on the liver. Due to the fact that Iranians frequently use *A. hirtifolium* in combination with yoghurt, it would be of great importance if such a study has been performed in humans with liver dysfunction.

## Figures and Tables

**Table 1:** Preliminary phytochemical screening of the *Allium hirtifolium* extract.

Phytochemical constituents	Methanol extract
Flavonoids	+++
Phenols	+++
Alkaloids	−
Proteins	−
Terpenoids	+
Steroids	+
Saponins	+++
Anthraquinones	−
Amino acids	+
Tannins	+
Glycosides	++

Strongly positive (+++), moderately positive (++), slightly positive (+), and negative (−).

## Data Availability

The data supporting the findings of this study are available within the article.
